# Evaluation of alternative RNA labeling protocols for transcript profiling with Arabidopsis AGRONOMICS1 tiling arrays

**DOI:** 10.1186/1746-4811-8-18

**Published:** 2012-06-13

**Authors:** Marlen Müller, Andrea Patrignani, Hubert Rehrauer, Wilhelm Gruissem, Lars Hennig

**Affiliations:** 1 Department of Biology, Plant Biotechnology, ETH Zurich, Zurich, Switzerland; 2 Functional Genomics Center Zurich, ETH and University of Zurich, CH-8057, Zurich, Switzerland; 3 Department of Plant Biology and Forest Genetics, Uppsala BioCenter, Swedish University of Agricultural Sciences and Linnean Center for Plant Biology, PO-Box 7080, SE-75007, Uppsala, Sweden

**Keywords:** Transcript profiling, Arabidopsis, Microarray, AGRONOMICS1, Tiling array, RNA labeling

## Abstract

Microarrays are routine tools for transcript profiling, and genomic tiling arrays such as the Arabidopsis AGRONOMICS1 arrays have been found to be highly suitable for such experiments because changes in genome annotation can be easily integrated at the data analysis level. In a transcript profiling experiment, RNA labeling is a critical step, most often initiated by oligo-dT-primed reverse transcription. Although this has been found to be a robust and reliable method, very long transcripts or non-polyadenylated transcripts might be labeled inefficiently. In this study, we first provide data handling methods to analyze AGRONOMICS1 tiling microarrays based on the TAIR10 genome annotation. Second, we describe methods to easily quantify antisense transcripts on such tiling arrays. Third, we test a random-primed RNA labeling method, and find that on AGRONOMICS1 arrays this method has similar general performance as the conventional oligo-dT-primed method. In contrast to the latter, however, the former works considerably better for long transcripts and for non-polyadenylated transcripts such as found in mitochondria and plastids. We propose that researchers interested in organelle function use the random-primed method to unleash the full potential of genomic tiling arrays.

## Background

Transcript profiling has become a routine experimental approach in many fields of biology. One way to perform such profiling is to use DNA microarrays. For the model plant *Arabidopsis thaliana*, microarrays that probe the transcriptome have been used for more than ten years [1-8], and ATH1 arrays manufactured by Affymetrix have probably been used most widely [9]. Although ATH1 arrays proved to generate robust and reliable data, they lack probes for one third of the annotated Arabidopsis genes. For genome-wide profiling, researchers have started to use transcriptome arrays from other manufacturers or genome tiling arrays, which contain probes against the entire genome. Tiling arrays are not restricted to probe mRNA but also other transcripts such as sRNA (small RNA), tRNA (transfer RNA) and miRNA (micro RNA), and yield information on splicing. In Arabidopsis, tiling arrays have already identified many novel genes, intergenic non-coding RNAs and antisense transcripts [10-16]. Tiling arrays have also frequently been used in Arabidopsis epigenome profiling such as with chromatin-immunoprecipiation (ChIP-chip) (for review see [17]) and for detection of deletion mutations [18]. Another major advantage of tiling arrays is that they are not limited to current genome annotations but can be re-analyzed when new genome annotation information becomes available. In *Arabidopsis thaliana*, the Affymetrix Arabidopsis 1.0R tiling array was used in diverse applications ranging from transcript discovery to ChIP-chip [13,15,19-23]. An alternative Arabidopsis tiling array is the recently developed AGRONOMICS1 Affymetrix array [24]. The design of the AGRONOMICS1 array is similar to the Affymetrix Arabidopsis 1.0R array but lacks mismatch probes. Instead, it contains probes against both genome strands while the Affymetrix Arabidopsis 1.0R array probes only one strand. This allows strand-specific transcriptome profiling on single AGRONOMICS1 arrays and gives doubled probe density for epigenome profiling applications.

Since its release, AGRONOMICS1 arrays have been used for many experiments some of which have been published [25-27]. Initially, the *GeneChip©3’ IVT Express kit* (Affymetrix) was utilized to prepare samples for hybridization to AGRONOMICS1 arrays because this kit was also widely used to prepare samples for ATH1 arrays. Nevertheless, this method has some disadvantages, mostly because it is based on oligo-dT priming. Oligo-dT priming disfavors labeling of long transcripts. In addition, non-polyadenylated transcripts are labeled only poorly. Because transcripts in organelles usually lack polyA tails [28], oligo-dT-based labeling methods are not suitable for projects where expression data for plastidial or mitochondrial genes are needed. In addition, it has been reported that the use of T7 sequences in common oligo-dT priming protocols can cause artifacts on tiling arrays [29]. Labeling methods based on random priming at the reverse transcription step carry the potential to overcome the limitations of oligo-dT-based labeling [30-34].

Here, we tested an alternative labeling method based on random priming at the reverse transcription step and develop appropriate data analysis routines. The comparison of this and the previously used protocols revealed, in general, a very good agreement between fold change values from both methods. Considerable differences between both methods were observed for transcript signal estimates for long transcripts and for organellar transcripts, which both are only inefficiently labeled by oligo-dT priming. In both cases, expression estimates were much larger for the method based on random priming. In summary, we present an RNA labeling procedure together with an appropriate data analysis pipeline that can replace established oligo-dT based methods in projects where expression of plastidial or mitochondrial genes is of interest.

## Results and discussion

### Analyzing AGRONOMICS1 arrays based on the TAIR10 genome version

A major advantage of genome tiling microarrays is that they can accommodate changes in genome annotation. After the AGRONOMICS1 array was developed, the TAIR10 version of the Arabidopsis genome was released. We generated new TAIR10-based CDF (chip description format) files, which can be used to generate expression estimates from raw data (CEL files) not only for new but also for past experiments (Table 1). We compared expression estimates derived from a published data set from dark-grown and illuminated seedlings, which was based on labeling with the Affymetrix IVT Express kit [24], using the TAIR9- and the TAIR10-based CDF files, and as expected found very high agreement between both (Additional file 1: Figure S1). Because antisense transcripts have attracted much attention in recent years, we generated a second TAIR10-based CDF file that can be used to profile antisense transcripts. Note that originally most labeling protocols for Affymetrix expression arrays generated labeled aRNA (antisense RNA), which hybridizes with probes against the antisense strand. Nowadays, however, protocols generating labeled cDNA or labeled aRNA are both common. Table 2 summarizes usage of the new CDF files.

**Table 1:** Properties of custom-made CDF files for AGRONOMICS1 arrays

**CDF file**	**Genome version**	**# of probes (ath**^**1)**^ **+ control probes)**	**# of probe sets (ath + control probe sets)**	**# of genes probed(M + P)**^**2)**^	**Description**
*agronomics1_ TAIR9_gene*	TAIR9(2009)	1,246,484 (1,093,816 + 152,668)	30,608 (30,237 + 371)	29,920(33 + 67)	Gene-specific probe sets of all suitable probes from the antisense strand
*agronomics1_ TAIR10_gene*	TAIR10(2010)	1,626,780 (1,474,112 + 152,668)	31,027 (30,656 + 371)	30,466(35 + 85)	Gene-specific probe sets of all suitable probes from the antisense strand
*agronomics1_TAIR10_gene_sense*	TAIR10(2010)	1,397,929 (1,245,261 + 152,668)	30,757 (30,386 + 371)	30,174(36 + 84)	Gene-specific probe sets of all suitable probes from the sense strand

^1)^ ath probes, probes against Arabidopsis sequences.

^2)^ M, mitochondrial genes; P, plastidial genes.

**Table 2:** Matching CDF files and RNA labeling protocols

**CDF file**	**3’-IVT kit**	**WT kit**
*agronomics1_ TAIR10_gene*	Detection of sense transcripts	Detection of antisense transcripts
*agronomics1_TAIR10_gene_antisense*	Detection of antisense transcripts	Detection of sense transcripts

**Figure 1:**
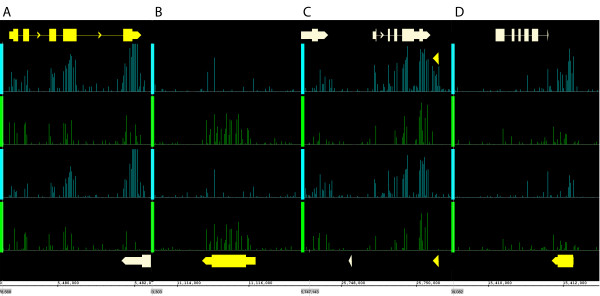
**Detection of antisense transcript signals on AGRONOMICS1 arrays. ** Bars represent gene annotations. Genes of interest are yellow, other genes grey. Probe signals from the *plus* strand are cyan and from the *minus* strand green. The upper two rows of signals show RNA from irradiated seedlings, the lower two rows RNA from dark-grown seedlings. **(A)***AT1G15950*, a gene on the *plus* strand. Signals are highest for probes from annotated exons and on the *plus* strand. **(B)***AT1G31130*, a gene on the *minus* strand. Signals are highest for probes on the *minus* strand. **(C-D)***AT5G64401***(C)** and *AT4G31875***(D)** are genes on the *minus* strand. However, signals are highest on the *plus* strand strongly suggesting antisense transcription. Note that the signals are specific for irradiated seedlings.

Applying the new CDF files to the dataset of RNA from dark-grown and illuminated seedlings, we identified 780 and 333 genes that were induced or repressed by light, respectively (p < 0.01, fold change > 2). With the same criteria, 5 and 0 genes had no significant difference in abundance of sense transcripts but had antisense transcripts that were induced or repressed by light, respectively. Three of the 5 genes overlapped with annotated genes on the opposite genome strand. Visual inspection of the tiling array data suggested that in these cases the apparent antisense signal was caused by a sense signal from the overlapping gene. In contrast, for two genes (*AT4G31875* and *AT5G64401*) strong antisense signals could not be explained by an overlap with known genes (Figure 1). Note that the IVT Express labeling protocol used here was earlier shown to have high strand-specificity [24]. Thus, the new antisense-CDF file can be used to quantify antisense transcripts.

The approach presented here differs from that of Coram and colleagues, who also quantified antisense transcript [35]. These authors used Affymetrix GeneChip Wheat Genome arrays, which are 3'IVT expression arrays and carry probes only for the sense strand. Therefore, two alternative labeling methods were used to label transcripts derived from sense or antisense transcription, respectively. Labeled samples were separately hybridized to arrays making two arrays per sample needed. It also required sufficient RNA for two labeling reactions per sample, which could be limiting for rare samples. In addition, the different labeling protocols imposed different sensitivities. In contrast, our approach relies on only one labeling reaction and hybridization and does not increase required amounts of RNA or experimental costs. Instead, labeled antisense transcripts are directly probed by complementary oligonucleotides present on the AGRONOMICS1 array. Because different probes are used to interrogate sense and antisense transcripts, signal intensities can also in this case not directly be compared. In most cases, however, such probe-specific effects will have a minor impact on the expression signals generated by RMA. It should also be noted that this approach approximates potential antisense transcripts based on the annotation of the sense transcript. To accurately determine transcript boundaries, algorithms to segment the hybridization signal along chromosomal coordinates are needed [36].

### Performance of the oligo-dT-based and random-primed labeling protocols

Aliquots from the same RNA preparations that were used previously with an oligo-dT-based protocol [24] were used for the random-primed protocol. Two technical replicates of a rosette leaf RNA sample and three technical replicates of a flower RNA sample were labeled and hybridized to AGRONOMICS1 tiling arrays. Table 3 shows the correlation among the technical replicates from the oligo-dT-based and the random-primed protocols. Although both protocols resulted in high data concordance, the random-primed protocol generated data with a slightly higher correlation.

**Table 3:** Correlation of probe set summary signals

	**oligo-dT primed**	**random primed**
*Index.R*		
Leaves	0.815 ± 0.023	0.861 ± 0.009
Flowers	0.889 ± 0.011	0.920
*c*_*Pearson*_		
Leaves	0.982 ± 0.003	0.977 ± 0.004
Flowers	0.962 ± 0.004	0.991

Mean and S.D. are listed for technical replicates. Correlation was expressed as *index.R* value [37] and as pair-wise Pearson correlation coefficient.

**Figure 2:**
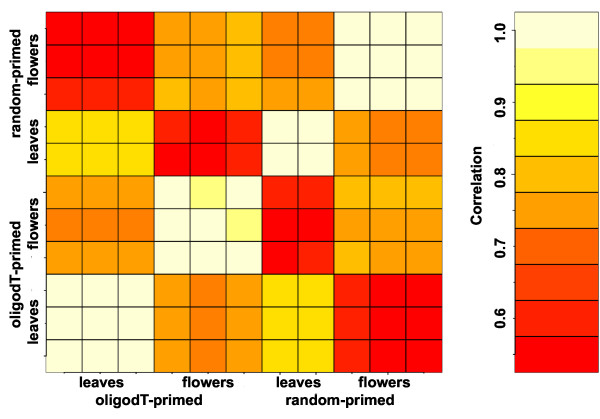
**Correlation of probe set summary values.** Pair-wise Pearson correlation is shown.

**Figure 3:**
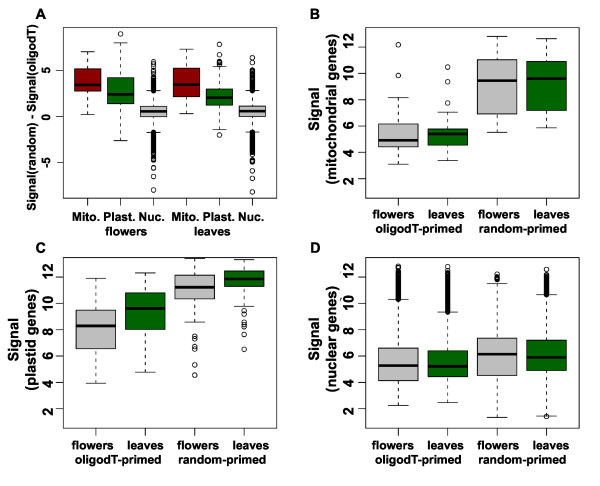
**Expression signals of organellar genes. (A)** Differences of signals from oligo-dT and random priming. Values larger than 0 indicate higher signals when using random priming. Mitochondrial, plastidial and nuclear genes were analyzed separately. Note that RMA-derived expression signals are in logarithmic scale. Therefore, differences in RMA-derived signals are equivalent to log2 of ratios (i.e. fold change) of signals in linear scale. **(B-D)** Expression signals in flowers (grey) and leaves (green) for mitochondrial **(B)**, plastidial **(C)** and nuclear genes **(D)**.

Next, we tested how well results based on the two labeling protocols correlated with each other. As evident from Figure 2, correlation between replicates of the same protocol was considerable higher than correlation between replicates of different protocols. This result suggested that array-based expression signals differed for many genes. Because transcripts in plastids and mitochondria are only polyadenylated as part of a polyadenylation-dependent RNA degradation mechanism [28], we hypothesized that expression signals for organellar genes would differ most between the two labeling protocols. Consistent with this hypothesis, there were mostly small differences between expression signals from nuclear genes while plastidial and in particular mitochondrial genes had consistently much higher expression signals when using the random-primed labeling protocol (Figure 3A). Expression signals of mitochondrial genes were independently of the protocol similar between flowers and leaves but about sixteen times larger when using the random-primed labeling protocol (Figure 3B). While signals were usually close to the detection limit when using the oligo-dT-primed method, many mitochondrial transcripts gave signals that were among the strongest in the genome when using the random-primed method. Expression signals of plastidial genes were independently of the protocol higher in leaves than in flowers. Nevertheless, these signals were about eight times larger when using the random-based labeling protocol and among the highest signals obtained on this array (Figure 3C). Signals for nuclear transcripts, in contrast, did not globally differ strongly between flowers and leaves, and also the labeling protocol had only a mild effect on these genes (Figure 3D).

**Figure 4:**
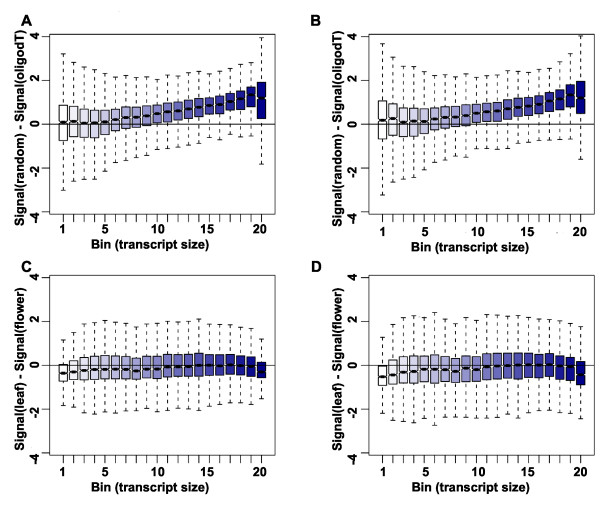
**Effect of gene length on expression signals. (A-B)** Differences of signals based on oligo-dT and random priming. Values larger than 0 indicate higher signals when using random priming. Nuclear genes were sorted into 20 bins according to transcript length. Signals from flower RNA **(A)** and leaf RNA **(B). (C-D)** Differences of signals from flowers and leaves. Values larger than 0 indicate higher signals in leaf RNA. Signals are based on oligo-dT **(C)** and on random priming **(D)**.

**Figure 5:**
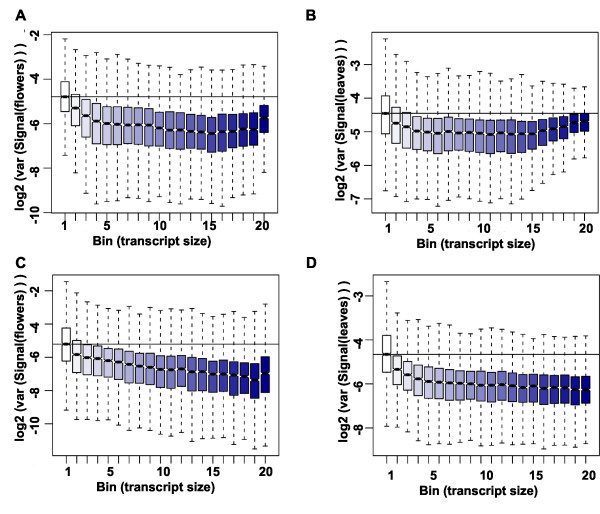
**Effect of gene length on expression variance.** Log2 of signal variance for oligo-dT **(A-B)** and random priming **(C-D)**. Nuclear genes were sorted into 20 bins according to transcript length.

In addition to organellar transcripts, also very long transcripts are expected to yield signals that differ particularly strongly between the labeling protocols, because multiple priming events in a random-primed protocol will generate more cDNA than single priming events in an oligo-dT-primed protocol. We tested this hypothesis by grouping the nuclear genes in 20 bins according to transcript lengths and plotting signal differences between protocols separately for each bin for flowers (Figure 4A) and leaves (Figure 4B). Indeed, signals for the ~25% shortest transcripts proved to be independent of the labeling protocol while signals for longer transcript were usually considerably larger when using the random-primed protocol. In contrast, differences in signal strength between leaves and flowers were independent of transcript length regardless of the labeling protocol (Figure 4C,D). These results show that expression signals are strongly affected by the labeling protocol, and thus direct comparisons of expression signals from experiments using different labeling protocols should be avoided. In contrast to expression signals, signal ratios did not strongly depend on transcript length (Additional file 1: Figure S2) suggesting that signal ratios can be compared even between experiments using different labeling methods. Because long transcripts can be interrogated by more probes than short transcripts, tiling array-based expression signals for long transcripts are expected to have higher precision than the signals for short transcripts. This effect is clearly visible in data based on oligo-dT priming for the shortest transcripts (Figure 5A,B). In contrast, for transcripts of intermediate length no such effect is evident, and for the longest transcripts signal variability even increases considerably. This increase in signal variability indicates variable labeling efficiency for long transcripts. In contrast, variability of expression signals based on random priming decreases over almost the entire range of transcript sizes (Figure 5C,D), suggesting that a major effect of reduced labeling efficiency of oligo-dT priming is increased measurement variability.

**Figure 6:**
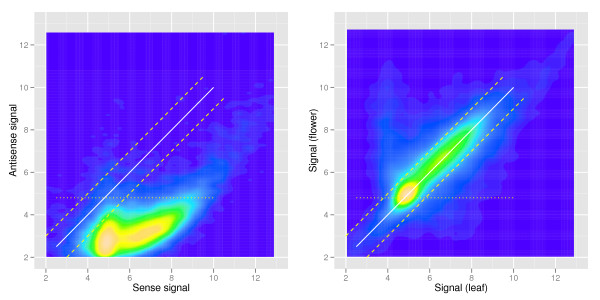
**Strand specificity of sample labeling. (A)** Leaf RNA was labeled using random priming and hybridized to AGRONOMICS1 arrays. Sense and antisense expression signals were calculated from the same CEL files using alternative CDF files. **(B)** Leaf and flower RNA was labeled using random priming and hybridized to AGRONOMICS1 arrays, and sense expression signals were calculated. Lines in (A) and (B) indicate: signal identity (x = y), solid lines; fold change of +2 and −2, dashed lines; empirical threshold for background signals, dotted lines.

Finally, we tested whether the used random-primed labeling method has sufficient strand-specificity to allow simultaneous detection of sense and antisense transcripts as shown above for data based on oligo-dT-primed labeling. Plotting sense and antisense signals for each gene revealed that even for genes with high sense transcript signals the antisense signal was usually very low (Figure 6A). For comparison, a plot of sense signals from leaf and flower samples showed a high correlation despite the great difference in tissue composition (Figure 6B). These results show that the used random-primed labeling method has a sufficient strand-specificity to justify quantification of antisense transcription.

Together, we found that the random-primed labeling protocol performed similar to the oligo-dT-primed protocol in most comparisons, but was more sensitive for organellar and long transcripts.

## Conclusions

Genomic tiling microarrays are valuable tools for biology, and we have developed two extensions that expand the application range of AGRONOMICS1 tiling arrays. First, we developed new CDF files for these arrays. Because the CDF files are based on the latest Arabidopsis genome version (TAIR10), estimation of gene expression levels will be more reliable. Importantly, raw data from past experiments can easily be re-analyzed with the new files. The AGRONOMICS1 tiling array contains probes from both genome strands, and we developed CDF files that contain probes located within annotated exons and match either the sense or the antisense strand. The CDF files can be used to simultaneously estimate levels of sense and antisense transcripts without the need for additional experiments or array hybridization.

Second, we tested an alternative labeling protocol that is not based on oligo-dT priming. Oligo-dT-based labeling methods are reliable and widely used for transcript profiling, but they suffer from certain deficiencies. In particular, oligo-dT priming fails to efficiently label organellar and very long transcripts. We found that when using AGRONOMICS1 tiling arrays a random-primed protocol compares favorably to the conventional oligo-dT-primed protocol. First, reproducibility of technical replicates was similar or even higher for the random-primed protocol. In addition, signal log ratios did not globally differ between both labeling methods, indicating that overall results are consistent and comparable. In contrast to signal ratios, signal values were less similar between the two methods. Therefore, a direct comparison of expression values is only justified for one and the same labeling method. Second, expression signals for long transcripts were considerably higher when using random priming. This causes an improved signal-to-noise ratio specifically for long transcripts. Third, expression signals of organellar transcripts were detected with much greater sensitivity and greater precision when using random priming.

In summary, alternative CDF files or labeling protocols enable the utilization of AGRONOMICS1 tiling arrays to interrogate antisense transcripts, transcripts from organelles or transcripts of very long genes in addition to the commonly probed nuclear mRNAs.

## Methods

### Plant material and RNA extraction

RNA samples were as described [24]. Briefly, *Arabidopsis thaliana* accession Columbia-0 plants were grown on soil at 23 °C in a photoperiod of 16 h of light and 8 h of darkness. Leaves (no. 4 from 10–15 plants per sample) and flowers (stage 15; 20–25 per sample) were harvested after 10 and 25 d, respectively. Total RNA was isolated using the Qiagen Plant RNeasy MiniKit according to the manufacturer’s instructions.

### Microarray target preparation

#### Method 1. GeneChip© IVT express kit

Microarray target preparation with the GeneChip© IVT Express Kit (Affymetrix, Santa Clara, CA) was described before [24].

#### Method 2. GeneChip© whole transcript (WT) sense target labeling assay

The starting material was 1 μg of total RNA. Then, microarray target preparation with the GeneChip© Whole Transcript (WT) Sense Target Labeling Assay (Affymetrix, Santa Clara, CA) was carried out as recommended by the manufacturer. Briefly, ribosomal RNA was removed using a RiboMinus™ Plant Kit (Invitrogen, Zug, Switzerland), which is not dependent on the polyadenylation status or the presence of 5'cap structure on the RNA. Then, random hexamers tagged with T7 promotor sequence are used to conduct a two-cycle cDNA synthesis following.

#### Array hybridization

Biotin-labeled microarray target samples were mixed in 300 μl of Hybridization Mix (Affymetrix) containing Hybridization Controls and Control Oligonucleotide B2 (Affymetrix). Samples were hybridized onto Affymetrix AGRONOMICS1 Arabidopsis tiling array for 16 h at 45 °C. Arrays were then washed using an Affymetrix Fluidics Station 450 following the FS450_0004 protocol. An Affymetrix GeneChip Scanner 3000 was used to measure the fluorescence intensity emitted by the labeled target.

### Generation of CDF files

Custom-made CDF files were generated as described [24]. Briefly, probes were mapped to the TAIR 10 genome sequence, and only probes with a single match inside an annotated exon (excluding untranslated regions) were used. Probe sets were generated if at least three such probes existed for a gene. For genes with multiple transcripts with little overlap, more than one probe set was generated per gene (see Table 4). The CDF file contains three types of probe sets, which can be discriminated by their names. The naming scheme is < locus name > . < variant > . < chromosome > . < strand > . < mRNA_start > . < mRNA_end > (e.g. AT1G01010.0.Chr1.plus.3631.5899). The meaning of the variant component is as follows: 0, there is only one transcript annotated for the gene, and the probe set matches this transcript; X, there are multiple transcripts with a large overlap annotated for the gene, and the probe set matches the intersection of all these transcripts; [1,N], there are multiple transcripts with little overlap annotated for the gene, and each probe set contains all probes that match the corresponding. Finally, there is a number of overlapping genes annotated in the genome for which no gene-specific probes sets could be formed. Thus, 94 probe sets were included that probe more than one annotated gene (90 probe 2 genes, 2 probe 3 genes, 2 probe 4 genes; e.g. AT1G06149_AT1G06150.X.Chr1.minus.1867015.1873718).

**Table 4:** Summary of probe sets on custom-made CDF files for AGRONOMICS1 arrays

	***agronomics1_ TAIR9_gene***	***agronomics1_ TAIR10_gene***	***agronomics1_ TAIR10_gene_antisense***
Number of probe sets	*30,608*	*31,027*	*30,757*
Control probe sets	*371*	*371*	*371*
Probe sets for Arabidopsis genes	*30,237*	*30,656*	*30,386*
Probe sets for nuclear genes	*30,137*	*30,536*	*30,266*
Probe sets for plastid genes	*67*	*85*	*84*
Probe sets for mitochondrial genes	*33*	*35*	*36*
One annotated transcript uniquely probed	25,387	24,426	24,139
The intersection of a set of annotated transcripts uniquely probed	4,325	5,661	5,644
Several annotated transcript exist with little overlap;	0	90	160
1 is specifically probed			
Several annotated transcript exist with little overlap;	131	70	86
2 are specifically probed			
Several annotated transcript exist with little overlap;	54	36	44
3 are specifically probed			
Several annotated transcript exist with little overlap;	15	6	6
4 are specifically probed			
Several annotated transcript exist with little overlap;	7	1	4
5 are specifically probed			
Several annotated transcript exist with little overlap;	1	1	1
6 are specifically probed			
2 largely overlapping genes probed	0	90	85
3 largely overlapping genes probed	0	2	2
4 largely overlapping genes probed	0	2	2

### Data analysis

All analysis was performed in R 2.12.1 [38]. Visualization of tiling array data was done using the Integrated Genome Browser at http://igb.bioviz.org[39]. Library files and scripts are freely available as Supplemental Data, at http://www.agron-omics.eu/index.php/resource_center/tiling-array or upon request from the authors. Expression signals were extracted from CEL files using RMA [40] implemented in the aroma.affymetrix package [41] as described earlier [24]. For the comparison of labeling methods, only genes with unique probe sets in both CDF files were used. Quantile normalization as implemented in the limma package [42] was used for normalization of expression values to achieve consistency between arrays.

### Availability

Library files and scripts are freely available as Supplemental Data S1 at http://www.agron-omics.eu/index.php/resource_center/tiling-array and at www.slu.se/genetics/resources/agronomics1

## Competing interests

The authors declare that they have no competing interests.

## Authors’ contribution

LH and WG discussed and LH designed the experiments. AP performed the experiments. MM, HR and LH analyzed the data. MM, LH and WG wrote the manuscript. All authors read and approved the final manuscript.

## Supplementary Material

Additional file 1: **Figures S1.** Correlation of expression signals from TAIR9- and TAIR10-based CDF files. Correlation is measured with *index.R*. The axis label *sig1* denotes replicates of TAIR9, *sig2* replicates of TAIR10. F1, F2, F3 are the flower replicates; L1, L2, L3 are the leaf replicates. **(A)** oligo-dT priming, **(B)** random priming. **Figure S2.** Signal ratios are not affected by gene length. Signal log ratios (SLR) between flowers and leaves were calculated, and differences between the SLR values based on oligo-dT and random priming were plotted. Values larger than 0 indicate higher fold changes when using oligo-dT priming. Nuclear genes were sorted into 20 bins according to transcript length. Click here for file
