# Supplementary material for: Evaluation of alternative RNA labeling protocols for transcript profiling with Arabidopsis AGRONOMICS1 tiling arrays
Source: Plant Methods. 2012 Jun 13;8:18. doi: 10.1186/1746-4811-8-18 (PMC3418198; doi:10.1186/1746-4811-8-18)
Supplement: Additional file 1: — Figures S1. Correlation of expression signals from TAIR9- and TAIR10-based CDF files. Correlation is measured with index.R. The axis label sig1 denotes replicates of TAIR9, sig2 replicates of TAIR10. F1, F2, F3 are the flower replicates; L1, L2, L3 are the leaf replicates. (A) oligo-dT priming, (B) random priming. Figure S2. Signal ratios are not affected by gene length. Signal log ratios (SLR) between flowers and leaves were calculated, and differences between the SLR values based on oligo-dT and random priming were plotted. Values larger than 0 indicate higher fold changes when using oligo-dT priming. Nuclear genes were sorted into 20 bins according to transcript length. [file 1746-4811-8-18-S1.pdf]

**Supplemental information**

**Supplemental figures**

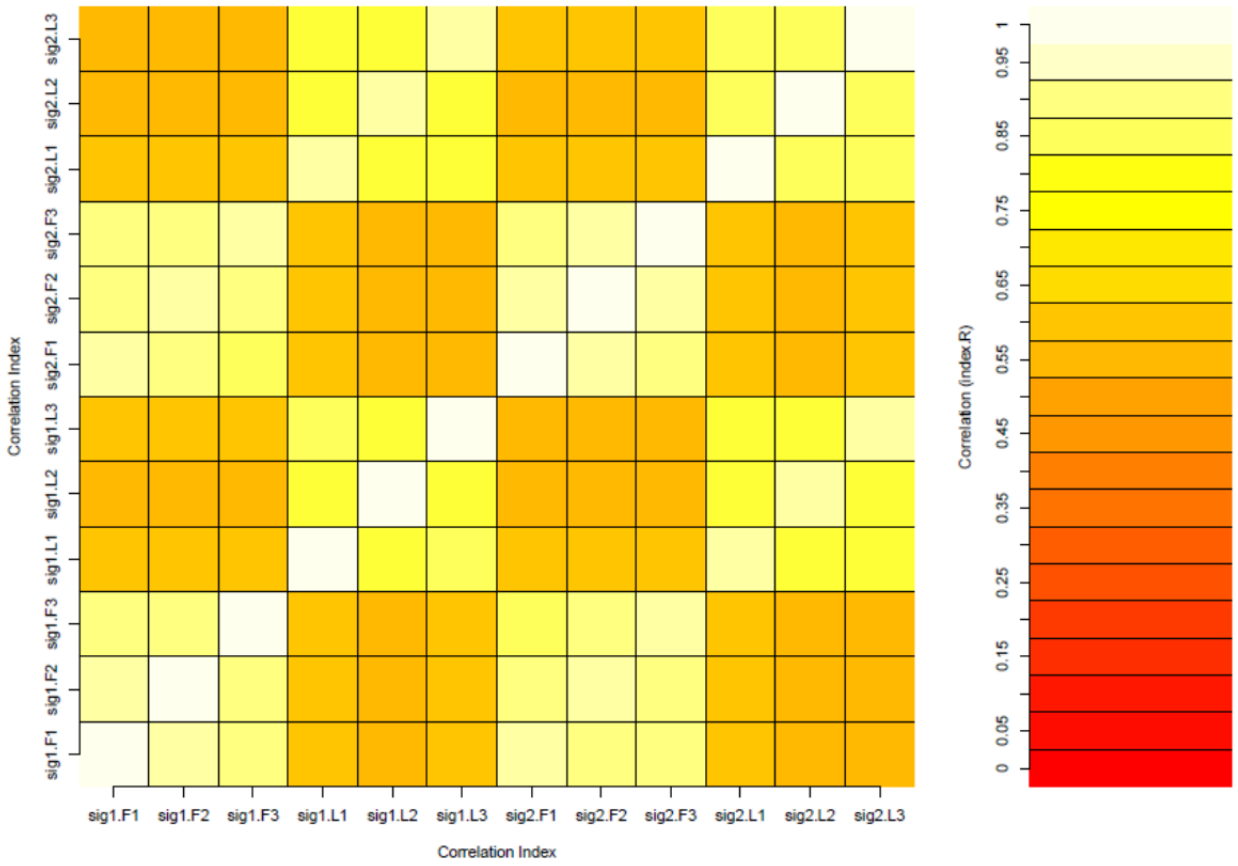


**Figures S1. Correlation of expression signals from TAIR9- and TAIR10-based CDF files.** Correlation is measured with index.R. The axis label *sig1* denotes replicates of TAIR9, *sig2* replicates of TAIR10. F1, F2, F3 are the flower replicates. L1, L2, L3 represent the leaf replicates. **(A)** oligo-dT-priming, **(B)** random priming.

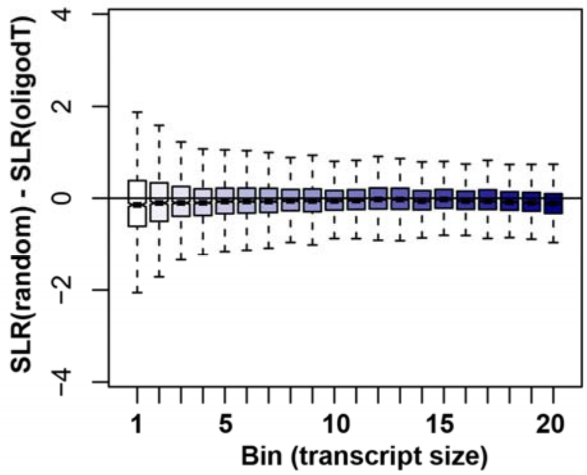


**Figure S2 Signal ratios are not affected by gene length.** Signal log ratios (SLR) between flowers and leaves were calculated, and differences between the SLR values based on oligo-dT- and random-priming were plotted. Values larger than 0 indicate higher fold changes when using oligo-dT priming. Nuclear genes were sorted into 20 bins according to transcript length.
